# T-cell exhaustion and stemness in antitumor immunity: Characteristics, mechanisms, and implications

**DOI:** 10.3389/fimmu.2023.1104771

**Published:** 2023-02-20

**Authors:** Xiaoxia Chi, Shahang Luo, Peng Ye, Wei-Lun Hwang, Jong-Ho Cha, Xiuwen Yan, Wen-Hao Yang

**Affiliations:** ^1^Affiliated Cancer Hospital & Institute and Key Laboratory for Cell Homeostasis and Cancer Research of Guangdong Higher Education Institutes, Guangzhou Medical University, Guangzhou, Guangdong, China; ^2^Department of Infectious Diseases, Guangzhou Panyu Central Hospital, Guangzhou, Guangdong, China; ^3^Department of Biotechnology and Laboratory Science in Medicine, National Yang Ming Chiao Tung University, Taipei, Taiwan; ^4^Cancer Progression Research Center, National Yang Ming Chiao Tung University, Taipei, Taiwan; ^5^Department of Biomedical Science, College of Medicine, and Program in Biomedical Sciences and Engineering, Inha University, Incheon, Republic of Korea; ^6^Graduate Institute of Biomedical Sciences, China Medical University, Taichung, Taiwan

**Keywords:** exhaustion, stemness, T_SCM_, chronic antigenic stimulation, cancer immunotherapy

## Abstract

T cells play a crucial role in the regulation of immune response and are integral to the efficacy of cancer immunotherapy. Because immunotherapy has emerged as a promising treatment for cancer, increasing attention has been focused on the differentiation and function of T cells in immune response. In this review, we describe the research progress on T-cell exhaustion and stemness in the field of cancer immunotherapy and summarize advances in potential strategies to intervene and treat chronic infection and cancer by reversing T-cell exhaustion and maintaining and increasing T-cell stemness. Moreover, we discuss therapeutic strategies to overcome T-cell immunodeficiency in the tumor microenvironment and promote continuous breakthroughs in the anticancer activity of T cells.

## 1 Introduction

When the body is exposed to antigenic stimulation (such as that from an infection or tumor), the immune system learns to recognize antigens, generates an immune response, and forms immune memory. Immunotherapy can effectively remove pathogens and tumor cells from the body by activating or mobilizing patients’ own immune system and subsequently restarting and maintaining attacks on pathogens and cancer cells (1, 2). Currently, cancer immunotherapy is proven to prolong the survival of patients (3). However, cancer immunotherapy is beneficial only for specific populations. Thus, cancer immunotherapy is among the most widely researched topics in oncology in order to uncover methods to improve its efficacy. With the popularization of cancer immunotherapy (4), T cells in the tumor microenvironment (TME) have received increasing attention because of their powerful immune effects. T-cell therapy is a promising antitumor immunotherapy that can reduce tumor progression (5, 6). Adoptive T-cell transfer (ACT) immunotherapies currently used in cancer immunotherapy include chimeric antigen receptor (CAR) T-cell therapy (7, 8), tumor-infiltrating lymphocyte (TIL) therapy (9), and engineered T-cell receptor (TCR) T-cell therapy (10). Although T-cell therapy resulted in high response rates in nonsolid tumors and sustained clinical remission in clinical trials (11, 12), a significant reduction in antigen-specific T cells can impair the ability of T cells to kill infected or tumor cells, resulting in limited immune response (13).

T-cell exhaustion, a state of effector T-cell dysfunction, was originally identified by Moskophidis et al. (14). During chronic infection or cancer progression, with the continual stimulation of inflammatory factors or antigens, T cells gradually lose their effector function and memory T-cell characteristics, resulting in the body being unable to maintain a long-term durable and effective immune response (15). T-cell exhaustion is a major obstacle to developing effective cancer immunotherapy (16). Moreover, T-cell exhaustion in tumors is negatively related to the therapeutic efficacy of immunotherapy (17, 18). Immunotherapies based on T-cell exhaustion have been widely investigated and studied. Currently, immunotherapy used to inhibit effector T-cell exhaustion involves the blocking antibodies that target immune checkpoint molecules, including cytotoxic T-lymphocyte antigen 4 (CTLA4; ipilimumab and tremelimumab) and programmed cell death protein-1 (PD-1; pembrolizumab, nivolumab, and cemiplimab) and its ligand PD-L1 (atezolizumab, avelumab, and durvalumab). These monoclonal antibodies improve antigen-specific T-cell activity and persistence by blocking the critical negative regulators of its function; however, the emergence of immune-related adverse events limits their clinical benefits (19–21). Scientists are attempting to develop new strategies to target terminally exhausted T cells (T_EX_) and restore immune function (22).

Memory T lymphocytes (T_M_) play a key role in the immune response to cancer and infectious diseases, producing a rapid immune response upon repeated antigenic stimulation. On the basis of the cell phenotype, distribution location and function, T_M_ can be divided into different subsets, namely stem cell-like memory T cells (T_SCM_), central memory T cells (T_CM_), effector memory T cells (T_EM_), tissue-resident effector memory T cells (T_RM_) and terminally differentiated effector memory T cells (T_EMRA_) (23–25). T_SCM_ and T_CM_ are mainly located in secondary lymphoid organs such as lymph nodes, T_EM_ circulate throughout the body through the blood and lymph, T_RM_ reside in various peripheral tissues, and T_EMRA_ are present in blood and blood-rich tissues (e.g., the spleen, bone marrow, and lung) (25). T_SCM_ play a crucial role in promoting antitumor and immune reconstitution because of their enhanced stem cell-like self-renewal capacity, multidifferentiation potential, and long-term effector functions, and T_SCM_ have been applied in cancer therapy (26–28). T_SCM_ can serve as a reservoir of effector T cells with potent therapeutic properties. Antigen-specific T_SCM_ survived in tumor-bearing mice for a long time and differentiated into other T-cell subsets, which effectively removed targeted tumor cells and mediated a superior antitumor response (29). Given the role of T_SCM_ in the antitumor response, T_SCM_ can be used as host cells for CAR engineering to generate CAR-modified T cells carrying the T_SCM_-surface phenotype. Efficient derivation of CAR-T_SCM_ could overcome cancer-specific T-cell deficiencies and improve the efficacy of adaptive cancer immunotherapy (30–32).

Currently, the limitations of T-cell therapy include low response rates, poor persistence, and proliferative exhaustion (33–35). Although T-cell exhaustion and T_SCM_ were first identified in infectious diseases, their mechanism and functions in tumors are also vital for the development of antitumor immunotherapy. In clinical treatment, a clear understanding of the effect of T-cell exhaustion and stemness on tumors can help improve and promote T cell-based therapy, thus contributing to the rational design of new treatment strategies. Therefore, in this review, we discuss the mechanisms underlying T-cell exhaustion and stemness and the latest improvements in T-cell adoptive transfer therapies based on these mechanisms. This review provides new insights that can be beneficial for the further study of adoptive T-cell therapy and its related clinical trials, which may contribute to the design and implementation of new therapeutic strategies.

## 2 T-cell exhaustion

### 2.1 Concept of T-cell exhaustion

During chronic infection or cancer, antigens or inflammatory factors of pathogens or tumor cells stimulate T cells for a prolonged period, resulting in the gradual loss of the function of memory and effector T cells. This process is called T-cell exhaustion (15). When naïve T cells are activated by antigens, costimulation, or inflammation, they exponentially proliferate and differentiate into effector T-cell subsets within 2 weeks (36). After the alleviation of inflammation or the clearance of antigens, most effector T cells die, and a small proportion of activated T cells transform into memory T cells. Upon repeated stimulation, these memory T cells rapidly reactivate and perform effector T-cell functions (36). However, upon continual stimulation, effector T cells gradually lose their ability to produce an immune response and the memory phenotype, thus becoming functionally exhausted (36). Cytokine changes are a key component of the exhaustion phenotype. IL-2, interferon-γ (IFN-γ), and tumor necrosis factor-α (TNF-α) secretions gradually decrease during T-cell exhaustion. IL-2 is an essential cytokine for T-cell survival and the activation of effective cell-mediated immune responses to infection and tumor. Sustained IL-2 production is maintained in an antigen-rich environment, which enables T cells to harness a robust antigen response (15, 37). The definition of T_EX_ remains controversial. The term was first used in the field of viral immunology where researchers observed exhaustion in CD8^+^ T cells during chronic lymphocytic choriomeningitis viral infection (14). This concept was later applied to the field of oncology. Exhaustion of T cells does not mean that T cells completely lose their function. Although T_EX_ are observed to be completely devoid of effector function in many tumors (38), they still produce certain effector moieties, including inflammatory cytokines and granzymes (15). These inflammatory cytokines and granzymes can still play a role in killing tumor cells. T-cell exhaustion is an adaptation to chronic antigenic stimulation and prevents damage caused by an excessive immune response (39). Therefore, whether T_EX_ are beneficial or harmful remains to be determined.

### 2.2 Biological characteristics of T-cell exhaustion

T_EX_ are functionally distinct from effector and memory T cells. T_EX_ exhibit persistently elevated expression levels of inhibitory receptors (IRs), such as PD-1, T-cell immunoglobulin and mucin domain 3 (TIM-3), lymphocyte activation gene 3 (LAG-3), CTLA4, and T-cell immunoreceptor with immunoglobulin and ITIM domains (TIGIT), and alterations in cytokines, epigenetic and transcriptional profiles, and metabolic patterns (15, 40, 41).

#### 2.21 Elevated expression of IRs

Sustained high expression of multiple IRs is a hallmark of T_EX_. However, IRs can be expressed on effector T cells. Many IRs are consistently highly expressed on T cells, including PD-1, TIM-3, LAG-3, CTLA4, and TIGIT, which can negatively regulate T-cell function and properties (40). For example, a study including Korean patients with liver cancer reported that T cells with high PD-1 expression exhibited higher expression levels of exhaustion-related genes and produced low levels of type I IFN and TNF (42). Another study indicated that TIM-3 may cause CD8^+^ T-cell exhaustion, resulting in decreased production of T-cell killing factors, namely perforin and granzyme B (43). LAG-3 maintains an exhaustion-like pattern in CD8^+^ T cells, and LAG-3 deficiency can enhance CD8^+^ T-cell effector-like function (44). Another breast cancer study revealed that CTLA4 can negatively regulate CD8^+^ T-cell function (45). In colorectal cancer, TIGIT promotes CD8^+^ T-cell exhaustion, leading to poor prognosis (46). Under normal conditions, upon the clearance of antigens, IR expression can weaken T-cell activation and reduce autoimmune damage caused to the body. The expression level of IRs on T cells is low during normal conditions but high during chronic infection and cancer (47). Coexpression of multiple IRs has been observed in many animal models and human studies (48–51). To some extent, the degree of IR coexpression reflects the severity of T-cell exhaustion (52). IRs can regulate T-cell function through three mechanisms: (1) IRs sequester target receptors or ligands through ectodomain competition (53), (2) IRs decay signals from activated receptors (such as TCR and costimulatory receptors) by regulating intracellular mediators (54), and (3) IRs induce inhibitory gene expression (55). In conclusion, T-cell exhaustion is often accompanied by elevated IR expression.

#### 2.2.2 Cytokines and T-cell exhaustion

Several inhibitory cytokines are present in the TME, including interleukin (IL)-10 and transforming growth factor-beta (TGF-β), both of which promote the generation of T_EX_ (56). IL-10 is a STAT3-inducing cytokine that is often associated with attenuated T-cell activation (57). T-cell exhaustion can be prevented and reversed by blocking IL-10 (58, 59). IL-10 is produced by various immune cell types, such as dendritic cells, B cells, monocytes, CD8^+^ T cells, and nonregulatory CD4^+^ T cells (60). IL-10 can inhibit the antitumor activity of tumor cells and promotes T-cell exhaustion in breast cancer and B16 melanoma models (61). IL-10 may act directly on T cells through STAT-3, indirectly through the regulation of APC, or both (60). In a glioblastoma model, a study found that IL-10-driven T-cell exhaustion can be rescued by JAK/STAT inhibition (62). Another report found that the simultaneous blockade of the IL-10 and PD-1 pathways in mice synergistically reversed CD8^+^ T cell-exhaustion and enhanced viral control, which suggests that IL-10 plays a role in controlling CD8^+^ T-cell exhaustion (59). Another cytokine, TGF-β, attenuates or inhibits immune cell activation by activating downstream SMAD transcription (63). During acute infection, TGF-β acts as a negative regulator of effector function and upregulates the proapoptotic factor Bim by inhibiting the transcription factor T-box transcription factor 21 (T-bet) (64). TGF-β is often found to be highly expressed, and the TGF-β–SMAD2 signaling pathway is activated upon the formation of T_EX_ during chronic antigenic stimulation (64, 65).

Other cytokines present in the chronic antigenic environment can inhibit T-cell exhaustion. IL-2 is a key cytokine necessary for T-cell survival and activation, enhancing infection and tumor immune responses. IL-2 is used in cancer therapy to enhance T-cell function (66). The combination of IL-2 with PD-1 pathway blockade can exert synergistic effects, and it may be an attractive strategy for tumor immunotherapy (67). However, IL-2 may exert opposite effects, resulting in the expansion of regulatory T cells (Tregs) (68). In HIV infection, although IL-2 increases the number of CD4^+^ T cells, it has a weak effect on CD8^+^ T cells and viral replication (68–71). Determining how IL-2 therapy can be used to suppress T_EX_ while attenuating its effects on Treg expansion may help in modulating T-cell exhaustion in cancer. IL-21 is another γ chain cytokine similar to IL-2 that is mainly produced by follicular helper CD4^+^ T (Tfh) cells, thus directly promoting the expression of basic leucine zipper ATF-like transcription factor (BATF), which is involved in initiating or maintaining effector CD8^+^ T-cell function (72). In the absence of IL-21R signaling, CD8^+^ T cells fail to respond, suggesting that IL-21 plays a role in antagonizing effector T-cell exhaustion (73, 74). However, studies using IL-21 to treat T-cell exhaustion are scarce. The use of IL-21 to reverse T-cell exhaustion and enhance antitumor immunity may be a beneficial strategy. Other inflammatory cytokines, such as type I IFN, are associated with T-cell exhaustion. IFN-α/β are key proinflammatory cytokines that can directly induce antiviral activity and activate innate immunity (75). IFN-α/β signaling can antagonize the exhausted T-cell progenitor pool by affecting the transcription factor T-cell factor 1 (TCF1), thus inhibiting T-cell exhaustion (76).

#### 2.2.3 Transcriptional regulation of T_EX_

**Figure 1:**
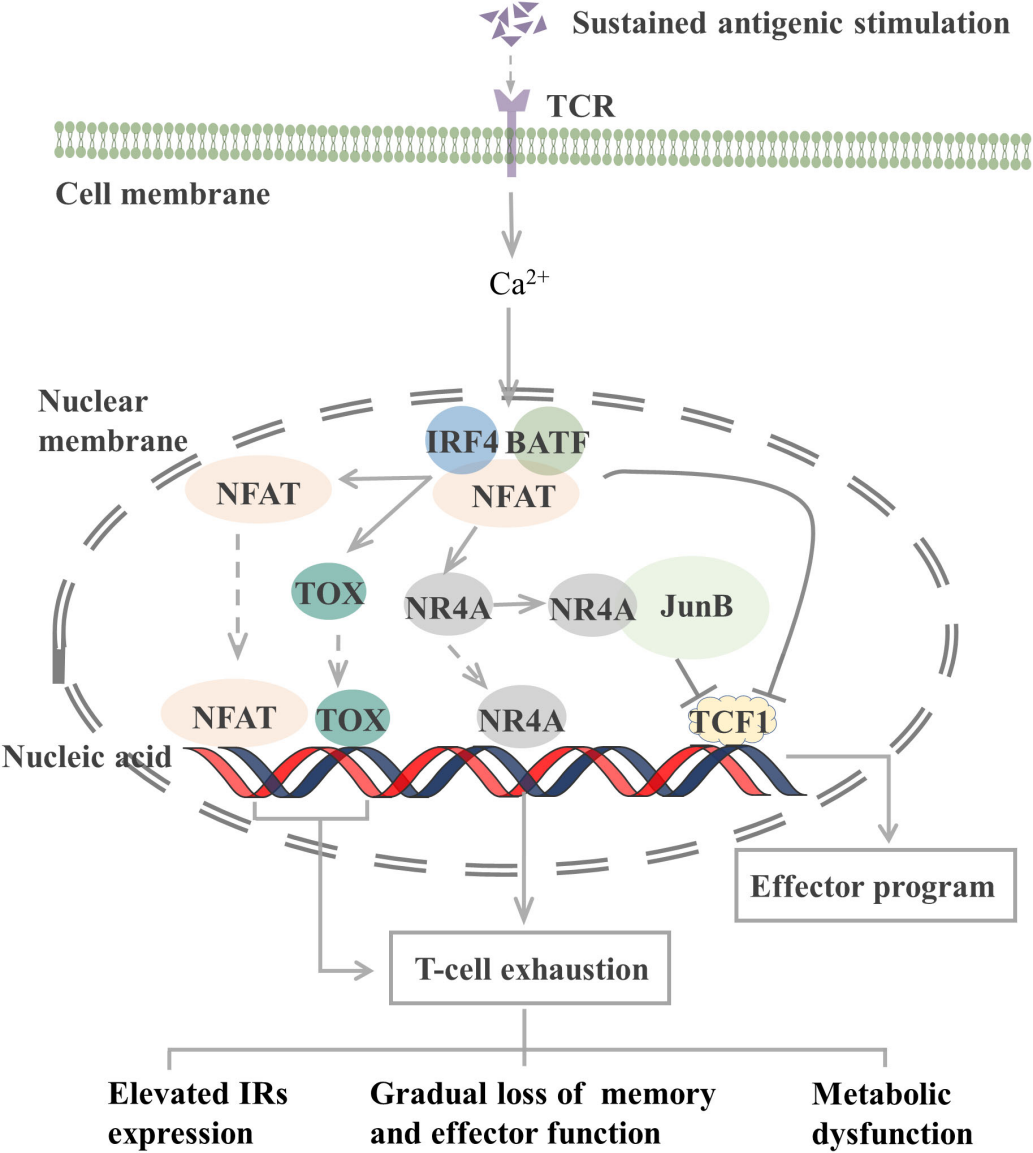
Transcription factors involved in the regulation of T cell exhaustion. The TCR signaling pathway activates the NFAT-BATF-IRF4 complex through calcineurin signaling, and the subsequent NFAT, TOX, and NR4A binding to the genome initiates T-cell exhaustion. When AP-1 is present, NR4A binds to it and represses TCF1 gene expression. In addition, the NFAT-BATF-IRF4 complex also inhibits TCF1 gene expression, which activates the effector function. T-cell exhaustion exhibits elevated IRs expression, metabolic dysfunction, and gradual loss of memory and effector function. TCR, T-cell receptor; NFAT, nuclear factor of activated T cells; BATF, basic leucine zipper ATF-like transcription factor; IRF4, Interferon regulatory factor 4; TOX, thymocyte selection-associated high mobility group box; NR4A, Nuclear receptor (NR) subfamily 4 group A; TCF1, T cell factor 1; IRs, inhibitory receptor. JunB, AP-1 transcription factor subunit.

The epigenetic characteristics of T_EX_ markedly differ from those of effector and memory T cells. Many transcription factors, including interferon regulatory factor 4 (IRF4), BATF, nuclear factor of activated T cells (NFAT), nuclear receptor subfamily 4 group A (NR4A), thymocyte selection-associated high mobility group box (TOX), and TCF1, which constitute a transcription factor regulatory network, are involved in T-cell exhaustion (**Figure 1**).

TCR-induced calcineurin signaling activates the transcription factors of the NFAT family and then interacts with other transcription factors, including activator protein-1 (AP-1), to control T-cell activation and effector cell differentiation. However, an elevated expression level of NFAT protein can drive CD8^+^ T-cell exhaustion (77). CD8^+^ T cells lacking NFAT fail to express exhaustion-related IRs. By preventing NFAT from interacting with AP-1, researchers determined that NFAT drives T-cell exhaustion by binding to sites in other genomic regions (77). Another study reported that the use of the calcineurin inhibitor FK506 to inhibit NFAT nuclear translocation and its transcriptional activity reduced the expression of IRs associated with T-cell exhaustion, including PD-1 and LAG-3, and increased the expression of TCF1, which is normally expressed on precursor exhausted T cells (T_PEX_, a population of T cells that maintain stemness) (78).

NFAT, together with BATF and IRF4, participates in a self-reinforcing transcriptional circuit, establishing a transcriptional network that promotes T-cell exhaustion during chronic infection and the transcription of *Pdcd1* to produce PD-1 (79). BATF is a member of the AP-1 family of transcription factors, and when it dimerizes with JunB, it can inhibit the activity of other AP-1s and promote T-cell exhaustion. Silencing of BATF reversed the function of T_EX_ in patients with HIV (55). IRF4 is a TCR signaling-sensitive transcription factor, and high IRF4 expression is a feature of T_EX_. IRF4 causes IR expression in T_EX_, impairs cytokine secretion, and inhibits anabolism. Low expression of IRF4 results in an overall increase in anabolism and a partial recovery of IFN-γ secretion (79). However, in a mouse tumor study, BATF and IRF4 synergistically shifted the phenotype and transcriptional profile of CAR-T cells from exhaustion to enhanced effector function, thus improving their antitumor response (80). In different microenvironments, transcription factors may exert different effects on T cells.

The NR4A family (orphan nuclear receptor family), including NR4A1, NR4A2, and NR4A3, and TOX are transcription factors downstream of NFAT that are involved in the development of T-cell exhaustion. When AP-1 protein is deficient, NFAT can activate TOX, TOX2, and NR4A families to induce the expression of IRs, such as PD-1, and inhibit the expression of effector molecules (81). Thus, the TOX and NR4A families are crucial transcription factors that limit the activity of effector T or CAR-T cells. CD8^+^ T cells express high levels of NR4As in tumors, and knockout of all three NR4As in CAR-T cells inhibited T-cell exhaustion, thus promoting tumor regression and prolonging survival in tumor-bearing mice (82). In the presence of AP-1, NR4A1 preferentially binds to AP-1 and inhibits its function to suppress effector gene expression. NR4A1 deletion inhibits T-cell exhaustion and enhances T-cell killing of tumors (83). Loss of TOX results in a reduction in the number of TCF1^+^ TIM-3^+^ progenitors that deplete CD8^+^ T cells. Knocking out TOX in mice reduced the expression of immune checkpoint molecules in immune cells and increased the expression of effector molecules (84). Taken together, both the NFAT-NR4A and NFAT-TOX pathways can cause T-cell exhaustion, which may be reversed by blocking these signaling.

#### 2.2.4 Metabolic programming of T_EX_

The metabolic programming of T_EX_ differs from that of effector or memory T cells. T_EX_ often exhibit metabolic dysfunction, such as respiratory chain inhibition, glucose uptake inhibition, and mitochondrial energy dysregulation (85). The PI3K, AKT, and mTOR signaling pathways play vital roles in T-cell development, function, and stability. When the inhibitory receptor PD-1 is upregulated in T cells, it suppresses the PI3K, AKT, and mTOR signaling pathways, leading to an exhausted T-cell state (86, 87). These metabolic disorders are associated with the increased expression of some IRs, such as CTLA4 and PD-1, which may exert inhibitory effects by regulating CD28 signaling (87–89). When PD-1 is blocked, the mTOR pathway is activated in T_EX_ and anabolic and glycolytic pathways are reactivated (90). In addition, tumor cell competition for glucose, oxygen, and other nutrients may promote T-cell exhaustion (40). Integrative omics analyses of liver cancer revealed the oncogenic reprogramming of hepatocellular carcinoma methionine recycling with elevated 5-methylthioadenosine and S-adenosylmethionine (SAM) expression to be closely linked to T-cell exhaustion (91). In conclusion, understanding the metabolic reprogramming of T-cell exhaustion can help in reversing the state of exhaustion and improving cancer immunotherapy.

### 2.3 “Stemness” in T-cell exhaustion

**Figure 2:**
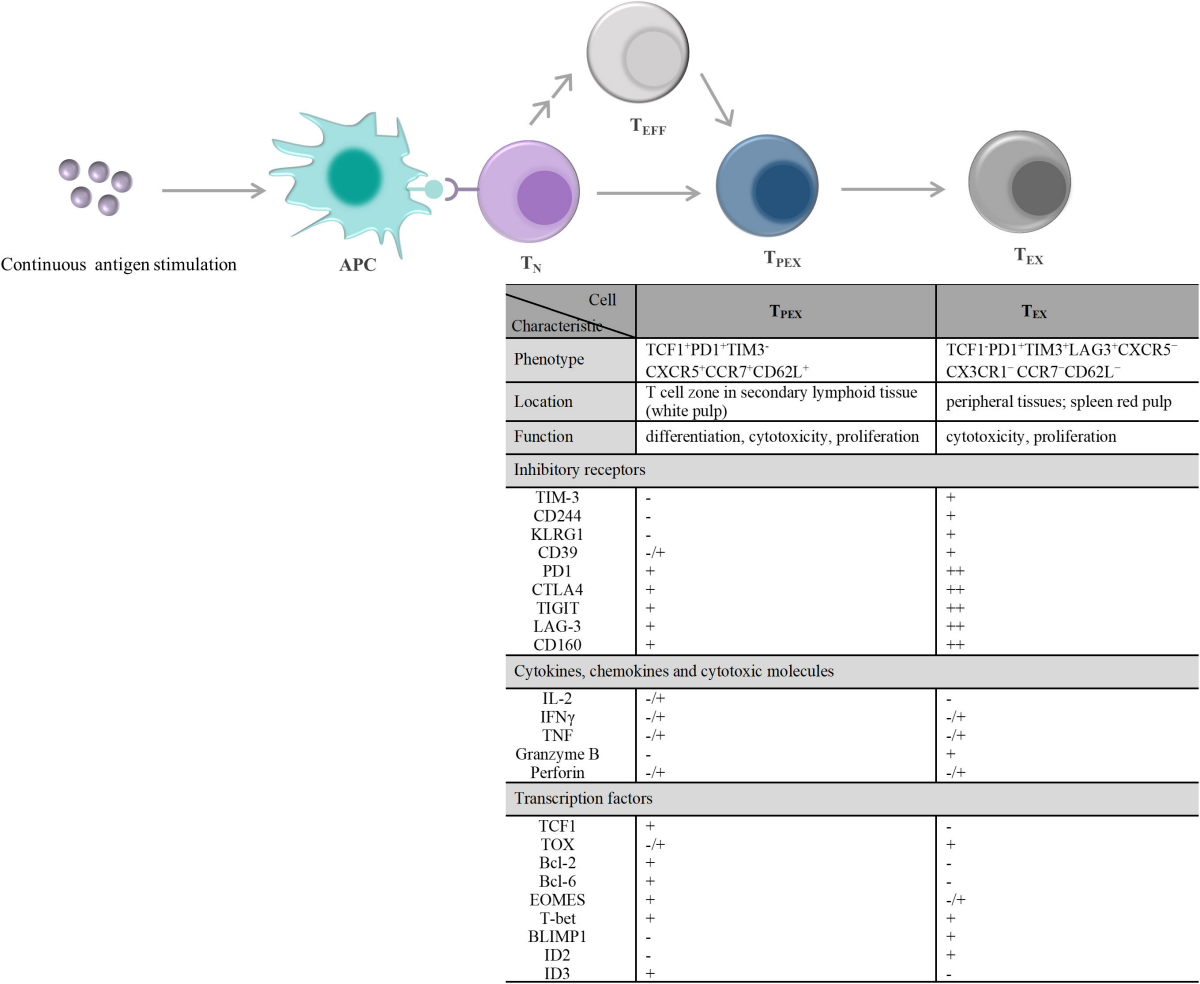
T cell exhaustion caused by continuous antigen stimulation and its alterations in the characteristic molecules. T_N_ exposure to persistently high amounts of antigen stimulation, including chronic infections and tumors. On the one hand, T_N_ differentiate into PD1^+^TCF1^+^ T_PEX_ cells, and then T_PEX_ continuously generate PD1^+^TCF1^–^ T_EX_ cells. On the other hand, T_EFF_ generated by T_N_ differentiation and/or produced by each stage of T cell differentiation also undergo the same T cell exhaustion. During exhaustion, there displayed a significant qualitative or quantitative difference about the inhibitory receptors expressed on the cell surface, the secretions of cytokines, chemokines, and cytotoxic molecules, and the transcription factors expression between T_PEX_ and T_EX_. - denotes “lack of expression”, **-/+** denotes “heterogeneous expression”, **+** and **+ +** denote “level of expression”. APC, antigen-presenting cell; T_N_, naïve T cells; T_EFF_, effector T cells; T_PEX_, precursor exhausted T cells; T_EX_, exhausted T cells; CTLA4, cytotoxic T lymphocyte antigen 4; TIGIT, T-cell immunoreceptor with immunoglobulin and ITIM domains; TIM-3, T-cell immunoglobulin and mucin domain 3; CXCR5, C-X-C motif chemokine receptor 5; CCR7, C-C motif chemokine receptor 7; PD1,programmed cell death 1; LAG-3, lymphocyte activation gene 3 protein; IL-2, interleukin-2; IFN, interferon; TNF, tumor necrosis factor; TCF1, transcriptional regulator T cell factor 1; TOX, thymocyte selection-associated high mobility group box; Bcl, B-cell lymphoma; EOMES, eomesodermin homologue; T-bet, T-box transcription factor; BLIMP1, B lymphocyte-induced maturation protein 1; ID, inhibitor of DNA binding.

T-cell exhaustion is a gradual development process. The exhausted T cell pool is heterogeneous and consists of two phenotypically and functionally distinct subpopulations, including T_PEX_ and T_EX_ (**Figure 2**). Compared with T_EX_, T_PEX_ are the class of stem cell-like subpopulations that maintain self-renewal, long-term proliferation, and differentiative capacity, which are beneficial for maintaining long-term antigen-specific T cell responses (92–94). T_PEX_ exhibit characteristics of an early exhausted phenotype, such as high expression of IRs (such as PD-1, LAG-3), and an impaired ability to secrete cytokines, IFN-γ, and TNF. However, T_PEX_ exhibit characteristics of stemness. T_PEX_ can perform self-renewal capacity and express factors associated with memory T cells, such as the transcription factors, TCF1 and BLIMP1 (92, 95, 96). T_PEX_ subsets have the potential to proliferate and differentiate into terminally exhausted effector T cells, which can continually replenish the terminally exhausted effector T cell pool to maintain T cell response function. It was reported that T_PEX_ have a more diversified TCR repertoire, and T_PEX_ differentiate into T_EX_ upon stimulation by a large number of antigens (92, 93). Recently, researchers found that the transcription factor MYB regulated the lifespan and limited function of T_PEX_, and revealed that ICB treatment-induced cytotoxic T cell proliferation burst and increased effector function depended on T_PEX_ and that increased T_PEX_ cell frequency is associated with increased survival (97). In general, adoptively transferred T_PEX_ mediates long-term inhibition of tumor growth and has become tumor therapeutic interventions (98, 99). In addition, T_PEX_ show similar characteristics to CD4^+^ follicular helper T cells, CD8^+^ memory precursor cells, and hematopoietic stem cell progenitors (98). T_PEX_ express the chemokine (CXC motif) receptor CXCR5 and the co-stimulatory molecule ICOS, which is beneficial for T_PEX_ to interact with B cells in lymphoid tissues and maintain its effector functions. Furthermore, T_PEX_ initiate a transcriptional program driven by TCF1/Bcl-6 to suppress T-cell exhaustion and maintain stemness characteristics, which suggests that this subset retains a long-lived memory potential. These regulations of T_PEX_ may be essential for persistent antigen-specific T-cell responses (76, 100, 101). These findings indicated that it is possible to develop therapies against chronic viral infections and cancer by T_PEX_ cells. As the central mediators of immune checkpoint blockade and T_EX_ pool replenishment in chronic antigen stimulation, T_PEX_ subsets can play key roles in the design of novel immunotherapeutic approaches. Thus, further understanding of T_PEX_ may improve the prognosis of cancer therapy.

## 3 T-cell stemness

With the progression of T-cell exhaustion, the therapeutic efficacy of antitumor immunotherapy becomes limited, resulting in persistent infections and leading to inefficient cancer control (102). Some T cells exhibit stem cell-like characteristics that are believed to be the key to triggering a robust, long-lasting antitumor response (27, 28, 103). By using the graft-versus-host disease mouse model, Zhang et al. identified a population of T cells expressing CD44^lo^CD62L^hi^ along with high expression of stem cell antigen-1, the antiapoptotic molecule B-cell lymphoma-2 (Bcl-2), and CD122 (IL-2 and IL-15 receptor β) in CD8^+^ T cells and named them T_SCM_ (104). Human T_SCM_ cells exhibited a CD45RA^+^CD45RO^-^CCR7^+^CD62L^+^CD27^+^CD28^+^CD127^+^CD95^+^ phenotype similar to that of T_N_ (CD45RA^+^CD45RO^-^CCR7^+^CD62L^+^CD27^+^CD28^+^CD127^+^ CD95^-^), and expressed high levels of CD122, CXCR3, CXCR4 and leukocyte function-associated antigen-1. T_SCM_ rapidly acquired effector functions upon TCR stimulation, which is relevant to the development of vaccines and T-cell therapy (28). Among all circulating T cells, T_SCM_ are a relatively small subset, accounting for only 2% to 3% of total T cells (28). T_SCM_ are characterized by their long lifespan and enhanced self-renewal and proliferation as well as their ability to replenish more differentiated memory and effector T-cell subsets (28, 104, 105). These characteristics make T_SCM_ a particularly attractive candidate for T-cell adoptive cancer immunotherapy. In particular, the strong stemness profile of T_SCM_ and their superior antitumor effect compared with those of other memory T lymphocytes make T_SCM_ potentially more suitable for immunotherapy (106). Therefore, we will mainly focus on the role of T_SCM_ in tumor immunity in this review.

### 3.1 Stem cell-like characteristics of T_SCM_

#### 3.1.1 Long lifespan

T_SCM_ can stably exist for a long time and survive longer than other memory subsets (105, 107). Genetically modified T_SCM_ can survive for decades in the human body while retaining their function and differentiation potential (108, 109). Pedro et al. reported the presence of dynamic heterogeneity in the human T_SCM_ pool. The T_SCM_ population consists of two subsets. The first subset includes the long-lived subset with dynamic properties required to maintain CD4^+^ and CD8^+^ memory T cells that have a half-life of approximately 9 years and a high degree of self-renewal. Another subset has a very short average clone life (half-life = 5 months) in the T_SCM_ pool. This high turnover rate causes difficulty in the formation of a stem cell-like population (110). The kinetic difference in the T_SCM_ pool may be related to naïve T-cell (T_N_) differentiation, which is affected by the type of antigen stimulation. Approximately 50% of T_SCM_ are differentiated from T_N_. Continual low-level exposure to novel environmental antigens and persistent antigens leads to continual T_N_ differentiation (110, 111). T_SCM_ are derived from thymic output (CD95^-^ T_N_) and cell proliferation induced by antigen stimulation (28). Due to age-dependent thymic degeneration, the number of T_N_ cells decreases, while the number of memory-differentiated T cells accumulates and the frequency of T_SCM_ cells is in a state of dynamic flux throughout the human lifespan (105, 112, 113). Interestingly, one study found that at advanced age and after the emergence of chronic inflammation, the Wnt/β-catenin pathway is impaired, and CD4^+^ T_SCM_ decrease (114). Therefore, future studies can determine how to characterize true T_SCM_ subsets, analyze the functions of the subsets, and determine their targeted induction.

#### 3.1.2 Enhanced proliferation

T_SCM_ exhibit a substantial proliferative capacity, which increases the proliferation and survival of T cells after xenotransplantation compared with naïve and traditional memory subpopulations, enhances antitumor activity, and eventually triggers tumor regression and cure (27, 28, 104, 115). Stem cells replicate through asymmetric division to promote diversified differentiation, meaning that one daughter cell maintains the characteristics of the original parent cell and the other daughter cell further differentiates into other types of cells (116). T cells undergo asymmetric division similar to that of stem cells. The intensity of the TCR signal during cell division is a key factor for regulating T-cell asymmetric division (117). T cells generate two different cell types through asymmetric division: the distal daughter and proximal daughter. The distal daughter retains long-term developmental plasticity, self-renewal, and differentiation capacity, similar to long-lived memory cells. The proximal daughter responds to antigen exposure (118–120). Memory T-cell subsets share the same ability to divide asymmetrically (33, 121, 122). T_SCM_ are a unique subpopulation of memory T cells. Therefore, T_SCM_ might divide in a similar manner to maintain a stable number, but the exact mechanism remains unexplored.

#### 3.1.3 Enhanced capacity for self-renewal and pluripotent differentiation

**Figure 3:**
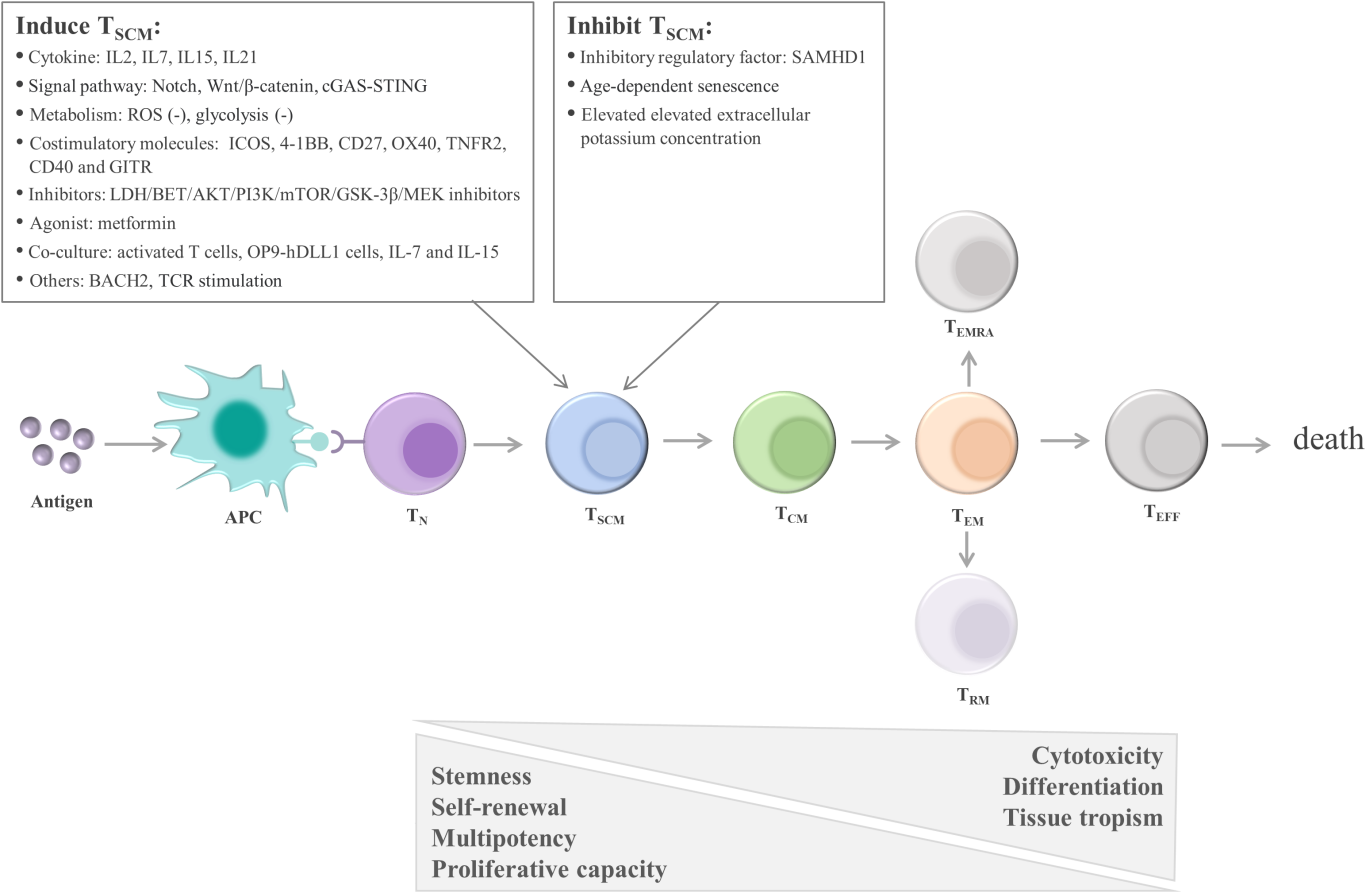
Linear lineage model and related regulatory factors in T_SCM_ cell differentiation. After exposure to antigens, T_N_ undergo proliferation, expansion, and differentiation. The differentiation process of T_SCM_ follows the known linear lineage model, that is, T_N_→ T_SCM_ → T_CM_ → T_EM_ → T_EFF_, eventually experiencing death. Furthermore, during T-cell exhaustion, T_PEX_ continuously replenish the terminally differentiated effector T_EX_. T_SCM_ are regulated by multiple factors, including cytokines, signaling pathways, metabolic factors, and costimulatory molecules. These factors regulate T_SCM_ mainly by promoting the transition of T_N_ to T_SCM_ and inhibiting the differentiation of T_SCM_. APC, antigen-presenting cell; T_N_, naïve T cells; T_SCM_, stem cell-like memory T cells; T_CM_, central memory T cells; T_EM_, effector memory T cells; T_EFF_, effector T cells; T_EX_, exhausted T cells; T_PEX_, precursor exhausted T cells; SAMHD1, SAM and HD domain-containing protein 1; IL, interleukin; ROS, reactive oxygen species; ICOS, inducible T-cell costimulatory; 4-1BB, TNF receptor superfamily member 9; OX40, TNF receptor superfamily member 4; LDH, lactate dehydrogenase; BET, bromodomain and extra terminal domain; AKT, serine/threonine kinase; PI3K, phosphatidylinositol 3-kinase; mTOR, mechanistic target of rapamycin kinase; GSK-3β, glycogen synthase kinase-3β; MEK, mitogen-activated protein kinase kinase; OP9‐hDLL1 cells, OP9 cells expressing Notch ligand, Delta‐like 1; BACH2, the transcription repressor BTB domain and cnc homolog 2; TCR, T-cell receptor.

One early study found that T_SCM_ are capable of self-renewal and differentiate into various memory T cells (115). Many studies support the linear lineage model of memory T-cell differentiation: T_N_→ T_SCM_ →T_CM_→T_EM_→ effector T cells (T_EFF_) (33, 123, 124) (**Figure 3**; **Table 1**). It is acknowledged that there is heterogeneity in each stage of T cell differentiation, and the cells in the differentiation process also undergo proliferation and differentiation upon antigenic stimulation and exert their memory or effector functions. For example, the lineage model was proposed based on the progressive differentiation of the stem-like memory T cell subpopulation in circulation, in which T_N_ first transform into memory T cells and then transition to effector T cells, thus the other T cell populations in peripheral tissues are not involved (25, 123). T_SCM_ represent the least differentiated long-lived memory T-cell subset with pluripotency to differentiate into multiple memory T-cell subsets (104, 107). T_SCM_ have the properties of traditional memory T cells, which can rapidly generate effector cytokines and enhance cellular immune effects upon secondary antigen stimulation, suggesting that T_SCM_ are the key to maintaining long-term immune memory (28). Therefore, the translational application of T_SCM_ in the development of new vaccines or adoptive T-cell therapy is promising. Most information on T_SCM_ has been obtained by studying CD8^+^ T cells. However, Muranski and colleagues reported that Th17 cells, a CD4^+^ T-cell subset, are a long-lived memory T-cell population with an enhanced self-renewal capacity. Th17 cells generate different Th lineages and retain stem cell-like molecular properties similar to early memory CD8^+^ cells through the coordination of HIF1α/Notch/Bcl-2 and promote long-term immunity (131–133). Adoptive transfer therapy of tumor antigen-specific CD4^+^ T cells during cancer immunotherapy is promising.

**Table 1:** The comparison between T_N_, T_SCM_, T_CM_, T_EM_, T_RM_, T_EMRA_, and T_EFF_.

Subset Characteristic	T_N_	T_SCM_	T_CM_	T_EM_	Terminal effector cells		
					T_RM_	T_EMRA_	T_EFF_
**Phenotype**	CD45RA^+^CD45RO^−^CCR7^+^CD62L^+^ CD27^+^CD28^+^ CD127^+^CD95^−^	CD45RA^+^CD45RO^−^CCR7^+^CD62L^+^ CD27^+^CD28^+^ CD127^+^CD95^+^	CD45RO^+^CD45RA^−^CCR7^+^CD62L^+^ CD28^+^	CD45RO^+^CD45RA^−^CCR7^−^CD62L^−^ CD28^+^	CD45RO^+^CD45RA^−^CCR7^−^CD62L^−^ CD69^+^CD103^+^S1P1^-^	CD45RA^+^CD45RO^−^CCR7^−^CD62L^−^ CD28^-^	CD45RO^+^CD45RA^−^ CCR7^−^CD62L^−^
**Location**	Blood, lymph nodes, spleen	Lymphoid tissue	Lymph nodes, spleen	Spleen, peripheral blood, bone marrow	Host tissue (including pancreas, stomach, kidney, and heart)	Blood and blood-rich tissues (spleen, bone marrow and lung)	Peripheral tissue
**Annotation**	Naïve	Stem-like memory	Central memory, naïve-like, memory	Effector memory, transitional	Tissue-resident memory, pre-exhausted	Effector memory recently activated, effector, cytotoxic	Effector, cytotoxic
**Transcription Factors**	Stemness-related genes: LEF1, TCF1, FOXO1, EOMES, ID3, Bcl-6, Bcl2, STAT3 gene expression gradually decreased. Differentiation-related and effector-related genes: T-bet, BLIMP1, STAT4, ID2, ZEB2 gene expression gradually increased.						
**Effectors after** **stimulation**	IL-2	IFN-γ, TNF, IL-2	IFN-γ, TNF, IL-2	IFN-γ, TNF, Perforin, IL-2, Granzyme B	IFN-γ, TNF, IL2, IL17, Granzyme B, TIA-1, HLA-DR, Ki67	IFN-γ, TNF, Perforin, Granzyme B	IFN-γ, TNF, Perforin, Granzyme B
**TCR diversity**	+	+/-	+/-	+/-	+	+	+
**Antigen specificity**	Antigen independence gradually decreased, while antigen addiction gradually increased.						
**Reference**	(25, 27, 125, 126)	(25, 27, 126)	(25, 27, 126, 127)	(25, 27, 125–127)	(24, 25, 27, 128)	(25, 27, 125, 129, 130)	(27, 101, 125)

-/+ denotes “heterogeneous expression”, + denotes “level of expression”.

### 3.2 Role of T_SCM_ in immune response and antitumor immunity

T_SCM_ are a double-edged sword in immune response. CD4^+^ T_SCM_ serve as a long-term viral reservoir for HIV type 1 (HIV-1) infection/latent infection, providing a stable host for HIV and functionally promoting long-term viral persistence in patients receiving highly active antiretroviral therapy (134). In adult T-cell leukemia (ATL), a T-cell malignant tumor caused by human T-cell leukemia type-1 (HTLV-1), the T_SCM_ population is susceptible to HTLV-1 infection. T_SCM_ are central to ATL initiation and maintenance and participate in ATL development by continually populating ATL clones (135). In addition, in autoimmune diseases, such as type 1 diabetes mellitus, systemic lupus erythematosus, immune thrombocytopenia, and rheumatoid arthritis, autoreactive T_SCM_ exhibit a high clonal expansion capacity and produce proinflammatory cytokines, leading to chronic autoimmune destruction and disease progression (136–139). In conclusion, the T_SCM_ pool might play a pathogenic role in T cell-mediated disease and contribute to disease maintenance and persistence. Thus, T_SCM_ can be a target for immunotherapy, and eradication of antigen-specific T_SCM_ can be the key to successful cures.

The long-term protective effect of T_SCM_ against acute and chronic infection has been demonstrated. Vaccination with virus-specific vaccines induced the production of T_SCM_, which can provide long-lasting immune protection (109). In addition, CD8^+^ T_SCM_ can control viral growth and enhance immune function, thus preventing the progression of chronic HIV-1 infection and improving clinical prognosis (140). In patients with chronic Chagas disease, CD8^+^ T_SCM_ were involved in filling the T-cell pool that controlled infection, and the number of T_SCM_ was negatively correlated with the severity of disease (141). The heterogeneous outcome resulting from these T_SCM_ is critical for immunotherapy, suggesting that clinicians should evaluate the immune effects of this subset on patients during treatment, understand the pathogenesis of the disease, and administer individualized treatment by specifically promoting or inhibiting T_SCM_, which may otherwise lead to an exacerbated immunosuppressive phenotype.

T_SCM_ exert strong and sustained antitumor effects (28, 142). Researchers have evaluated the effect of T-cell differentiation status (T_SCM_, T_CM_, and T_EM_) on tumor therapeutic efficacy. They found a significant negative correlation between T-cell differentiation status and antitumor efficacy *in vivo*. The lower the differentiation degree of T-cell subsets is, the more significant the antitumor effect is. The order of their therapeutic effect is T_SCM_ > T_CM_ > T_EM_ (106). Furthermore, in the mesothelioma mouse model, human-derived mesothelin-specific T_SCM_ exhibited enhanced antitumor activity (28). In the melanoma mouse model, T_SCM_ caused a sustained decrease in tumor growth, which was characterized by a significant increase in the overall survival rate (142). In the local lymph nodes of patients with non-small-cell lung cancer, CD4^+^ T_SCM_ were increased in number and function and produced IFN-γ, which may be related to sustained antigen stimulation or exposure to the TME. However, the mechanism by which such CD4^+^ T_SCM_ exert antitumor effects remains unclear (109). In cervical cancer caused by human papillomavirus (HPV) infection, HPV16-specific CD8^+^ T_SCM_ can induce long-term antitumor immunity and significantly inhibit tumor growth (143). The powerful immune reconstitution potential of T_SCM_ indicates that T_SCM_ can be useful in cancer treatment. Further exploration of T_SCM_ and their regulation may contribute to the design and clinical development of adoptive T-cell therapy and provide new directions for further improvement of adoptive T-cell immunotherapy.

### 3.3 Factors that promote T_SCM_ generation and enhance T_SCM_ stemness

#### 3.3.1 Cytokines

T_N_ can be differentiated into T_SCM_ *in vitro*. For example, stimulation with IL-2, IL-7, IL-15, and IL-21 promoted the enrichment and proliferation of tumor antigen-specific T_SCM_, which exhibited more distinct heterogeneous differentiation and self-renewal potential than did the original T_SCM_ isolated *in vivo* (144–147). In addition, lactate dehydrogenase inhibitors synergize with IL-21, thus promoting CD8^+^ T_SCM_ formation (148).

#### 3.3.2 Signal pathways

T_SCM_ phenotypes can be induced *in vitro* by suppressing genes related to T-cell differentiation, such as T-bet, BATF, and eomesodermin, and upregulating genes related to stemness, such as TCF1 and lymphoid enhancer-binding factor 1 (LEF1) (149). For example, the use of Notch signaling which induces the generation of antigen-specific T_SCM_ with enhanced antitumor effects (150), pharmacological inhibition of PI3K/Akt/mTOR signaling (115, 151–154), restriction of reactive oxygen species metabolism with antioxidant N-acetylcysteine (155), and targeted inhibition of the glycolytic pathway (156). Moreover, pharmacological inhibition of bromodomain and extraterminal domain protein results in the downregulation of BATF, which is necessary to initiate effector CD8^+^ T-cell differentiation (157), and subsequently inhibits further differentiation of T_SCM_ and improves T-cell persistence in the ACT immunotherapies (158). Moreover, weakening T-cell receptor signal intensity facilitates the formation of T_SCM_ with enhanced antitumor effects (145, 159).

TWS119, an inhibitor of glycogen synthase kinase-3β (GSK-3β), induces the activation of the Wnt/β-catenin signaling pathway. Subsequently, TWS119 inhibits CD8^+^ T-cell proliferation and blocks T_SCM_ cell differentiation by inducing the expression of TCF1 and LEF1, thus regulating the stemness of CD8^+^ T cells and promoting T_SCM_ cell generation. GSK-3β inhibitors/Wnt signaling agonists can be used as adjuvants to improve the stemness characteristics of T_SCM_ (29, 115, 160, 161). Inhibition of GSK-3β increases the cytotoxic capacity of CD8^+^ T_SCM_ and may indirectly mediate antitumor effects by enhancing their proliferation and differentiation into terminally differentiated CD8^+^ T cells (162). Moreover, cGAS-STING-driven type I IFN signaling promotes the differentiation of T_N_ into T_SCM_ subset and improves the efficacy of CAR-T therapy in mice by increasing TCF1 expression (163). Pearce et al. reported that metformin treatment activated AMP-activated protein kinase, promoting the generation of memory T lymphocytes in infection and tumors and thus enhancing protective antitumor immunity (164). In addition, MEK inhibitors increased T_SCM_ cell generation and antitumor effects by delaying T-cell division and enhancing mitochondrial biogenesis and fatty acid oxidation (165).

#### 3.3.3 Noncytokine factors

The costimulatory molecules ICOS, 4-1BB, and other T cell costimulatory molecules CD27, OX40(CD134), TNFR2, CD40 and GITR in TNFRSF (143, 166–169) and the transcription repressor BTB domain and CNC homolog 2 (BACH2) (170, 171), as the key regulators of stem cell-like T-cell subsets, induce the generation of stem cell-like lineages. Furthermore, the duration and intensity of the antigenic peptide/MHC-TCR interaction can affect T_SCM_ expansion and maintenance. CD8^+^ T cells transiently stimulated by CD3/CD28 antibodies have a more abundant T_SCM_ subset and exhibit greater cytokine production capacity (145, 159, 172). An appropriate TCR signal-stimulation intensity can induce T_SCM_ production to the greatest extent (173). Tumor antigen-specific T_SCM_ can be transformed from T_N_. In addition, activated T cells can be transformed into T_SCM_-like cells. Coculture of activated T cells with OP9-hDLL1 cells, IL-7, and IL-15 efficiently generated T_SCM_ with strong antitumor activity (174). The aforementioned findings demonstrate that T_SCM_ can be induced *in vitro* or *in vivo* and used to promote cancer immunotherapy. Therefore, exploiting guidance signals to induce the formation and expansion of T_SCM_ to supplement the effector T-cell pool represents a novel strategy to improve T cell-based tumor immunotherapy and enhance antitumor effects.

### 3.4 Factors associated with T_SCM_ inhibition

SAM and HD domain-containing protein 1 (SAMHD1), a deoxyribonucleoside triphosphate triphosphohydrolase, is an active restriction factor in CD4^+^ T_SCM_. During HIV-1 infection, a proportion of CD4^+^ T_SCM_ exhibit increased sensitivity to HIV-1 infection by expressing the HIV core receptors CCR5 and CXCR4. SAMHD1 exerts an inhibitory effect on CD4^+^ T_SCM_ by inhibiting viral replication and transcription in CD4^+^ T_SCM_ by hydrolyzing the active metabolite, nucleotide triphosphate (175). Moreover, the number of T_SCM_ in peripheral circulation markedly declines with age (176). During aging, the aggravation of inflammation and chronic infection affects the distribution of CD4^+^ T-cell subsets. Moreover, persistent stimulation can lead to the differentiation of CD4^+^ T_SCM_ into a proinflammatory state, with impaired signaling of genes related to the generation of T_SCM_, particularly the Wnt/β-catechin pathway, thereby disrupting the genetic signature and homeostasis of T_SCM_ (114). Future studies can identify other factors that impair T_SCM_ cell function and elucidate the regulatory mechanisms of T_SCM_ to promote or restore the function of T_SCM_. Therefore, Various factors related to T_SCM_ cell inhibition can be used as a target to improve the immune response and thus improve the efficacy of immunotherapy.

### 3.5 Other factors affecting T-cell stemness

As tumor cells die, K^+^ is released into the extracellular space, resulting in a substantial increase in potassium gradients in the TME. The elevated extracellular K^+^ concentration limits T-cell terminal effector differentiation while maintaining CD8^+^ T-cell stemness and enhancing antitumor function, suggesting that altered ion gradients in the TME are crucial for T-cell stemness and antitumor immunity (177, 178). Other immune and stromal cells also have been reported to affect T-cell stemness (179). The complex intercellular interactions in the immune system suggest the importance of these cells for the induction or maintenance of immune function in T_SCM_. However, current research in this field is scant, and future studies on this topic are warranted.

## 4 Targeting T-cell exhaustion and T_SCM_ to enhance antitumor immunity

### 4.1 Reverse T-cell exhaustion and restore tumor-infiltrating T cells in the TME

#### 4.1.1 Targeting immune checkpoint receptors

**Figure 4:**
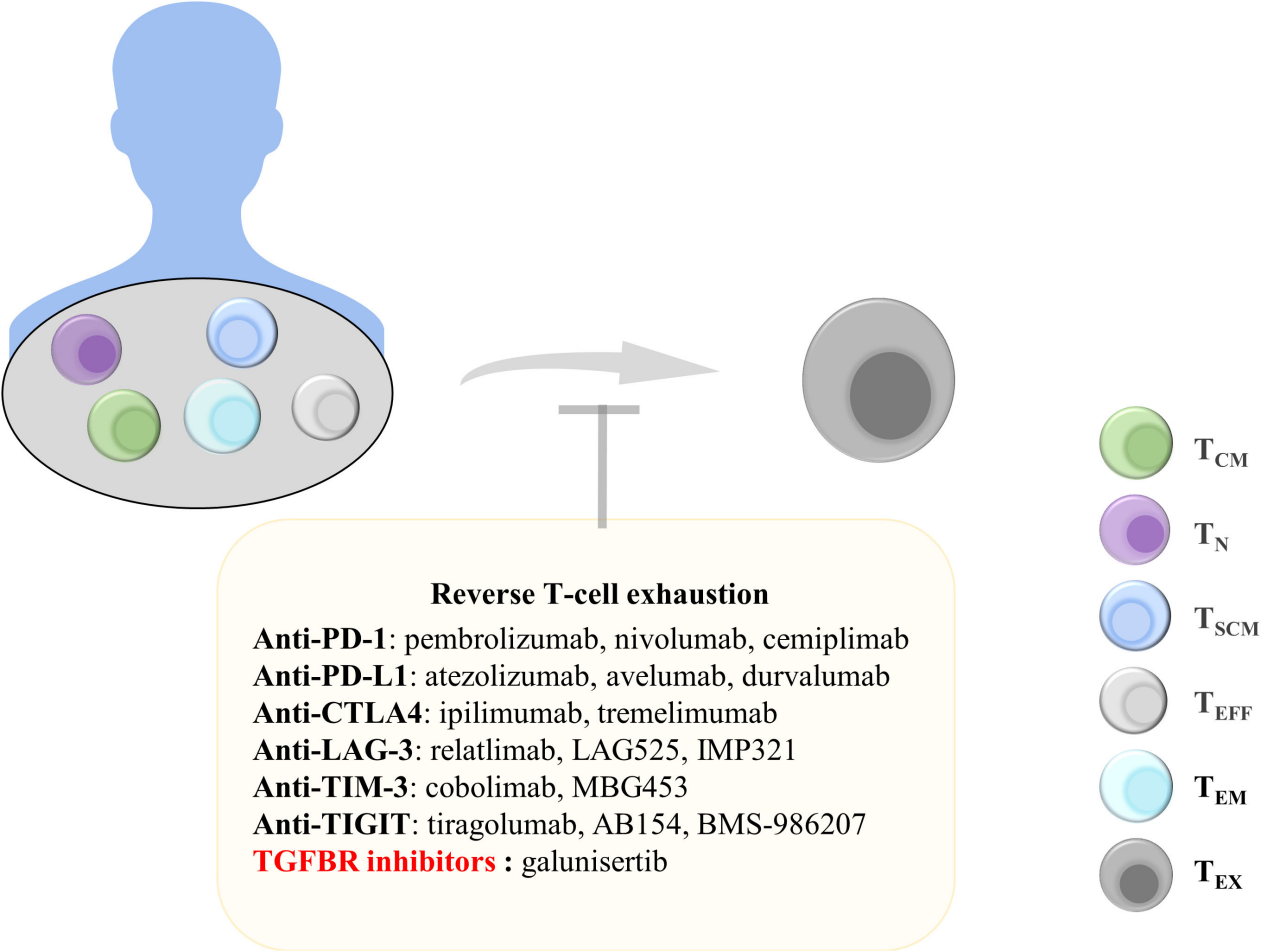
Treatments to reverse T-cell exhaustion. Reversing T-cell exhaustion by using anti-PD-1, anti-PD-L1, anti-CTLA4, Anti-LAG-3, Anti-TIM-3, Anti-TIGIT, and TGF-β inhibitors can prevent the generation of T_EX_. T_CM_, central memory T cells; T_N_, naïve T cells; T_SCM_, stem cell-like memory T cells; T_EFF_, effector T cells; T_EM_, effector memory T cells; T_Ex_, exhausted T cells; Anti-PD-1, anti-programmed cell death protein 1; PD-L1, programmed death ligand 1; CTLA4, cytotoxic T lymphocyte antigen 4; LAG-3, lymphocyte Activation Gene 3; TIM-3, T-cell immunoglobulin and mucin domain 3; TIGIT, T-cell immunoreceptor with immunoglobulin and ITIM domains; TGFBR, transforming growth factor-beta receptor.

High expression of TIL inhibitory receptors, which may be affected by the TME, leads to the increased expression of immune checkpoint receptors and promotion of T-cell exhaustion. T cells with the exhausted phenotype cannot produce effector factors to successfully target tumor cells, resulting in the decreased secretion of IL-2 and IFN-γ (46). T_EX_ characteristically express TIM-3, LAG-3, TIGIT, and PD-1 (46). Therefore, targeting these immune checkpoints may effectively alleviate T-cell exhaustion. Blocking the binding of PD-1 and its ligand PD-L1 and CTLA4 can significantly improve the killing function of T cells, and immune checkpoint inhibitor (ICB) monoclonal antibodies have been approved by the Food and Drug Administration for clinical use (180–182) (**Figure 4**).

PD-1 is an inhibitory receptor expressed on T_EX_. Blocking PD-1 and PD-L1 binding can promote the effector function of CD8^+^ T cells. A study examining peripheral blood samples from patients with advanced non-small-cell lung cancer receiving PD-1-targeted therapy reported that most patients exhibited an increase in the number of PD-1^+^CD8^+^ T cells after treatment and that this increase was correlated with treatment benefits (183). Nivolumab and pembrolizumab, which are PD-1 monoclonal antibodies, have been approved for melanoma treatment (184, 185). In addition, atezolizumab has been approved as a PD-L1 antibody for the treatment of uroepithelial carcinoma, and it exhibited significant therapeutic effects when used clinically (186). Tumor regression was observed in mice with pancreatic tumors that received a combination of an anti-PD-1 antibody and an OX40 agonist after 225 days. The combination therapy reduced the proportion of Tregs and T_EX_ in pancreatic tumors (187). In addition, the combined targeting of PD-1 and TIM-3 counteracted CD8^+^ T-cell exhaustion and restored T-cell function (188). Anti-PD-L1 antibodies can also block the binding of PD-L1 to PD-1, thereby inhibiting the exhaustion of T cells. Combination therapy with anti-PD-L1 and the tyrosine kinase inhibitor nilotinib can reduce T-cell exhaustion markers in leukemia and significantly improve the long-term survival rate of mice with leukemia (189). Anti-PD-1 and anti-PD-L1 therapies can reduce T-cell exhaustion and achieve superior treatment outcomes. Therefore, anti-PD-1 and anti-PD-L1 treatment strategies are still being explored to identify better therapeutic effects.

CTLA4 competes with CD28 to bind to B7 molecules; thus, T cells cannot receive costimulatory signals necessary for activation (190). CTLA4 is another valuable target for restoring T-cell exhaustion. The monoclonal antibody ipilimumab that targets CTLA4 is approved for the treatment of melanoma. The 5-year survival rate was significantly higher in patients with advanced melanoma receiving ipilimumab in combination with nivolumab than in patients receiving monotherapy (191). ICB use can result in satisfactory outcomes in patients with cancer. The discovery of additional inhibitory targets on T_EX_, including LAG-3 and TIGIT, can enable the development of targeted therapeutic strategies for tumors.

#### 4.1.2 Inhibitors of soluble mediators

Some cytokines play a key role in T-cell exhaustion. For example, the use of IL-2 or blocking IL-10 and TGF-β can effectively block T-cell exhaustion. IL-2 can downregulate transcription factors related to T-cell exhaustion. Moreover, IL-2 combined with PD-1 treatment can significantly inhibit T-cell exhaustion and increase the expression of effector molecules (192). Alternatively, PD1-IL2v, a new immunocytokine that overcomes the need for IL-2 receptor α-chain (CD25) binding by docking cis into PD-1, differentiates stem cell-like CD8^+^ T cells into T-cell subsets by using a better effector function (193). IL-10 is an inhibitory cytokine that promotes T-cell exhaustion, and IL-10 reduction can improve antitumor immune responses (194). In patients with advanced melanoma, using the anti-PD-1 antibody while inhibiting IL-10 enhanced the cytotoxicity of CD8^+^ TIL cells (195). In addition, combining the anti-IL-10 monoclonal antibody with Toll-like receptor 9 ligand CpG significantly improved antitumor efficacy (196). IL-10 inhibition can be an effective tumor treatment strategy. In addition, TGF-β is involved in T-cell exhaustion. Blocking TGF-β is another target for T-cell therapy, which has been included in clinical trials (197). Compared with PD-1 therapy alone, treatment with a combination of TGF-β inhibitors and the PD-1 antibody more significantly increased the number of CD8^+^ T cells in patients with myeloma (198). In addition, galunisertib, a selective TGF-β receptor (TGFBR) inhibitor, can be used to prolong overall survival in patients with unresectable pancreatic cancer (199). Inhibition of these cellular mediators in the TME can effectively inhibit T-cell exhaustion, indicating their crucial role in T cell-based cancer immunotherapy.

### 4.2 Enhancement and maintenance of T-cell stemness

#### 4.2.1 Enhancement of T-cell stemness-related gene expression

**Figure 5:**
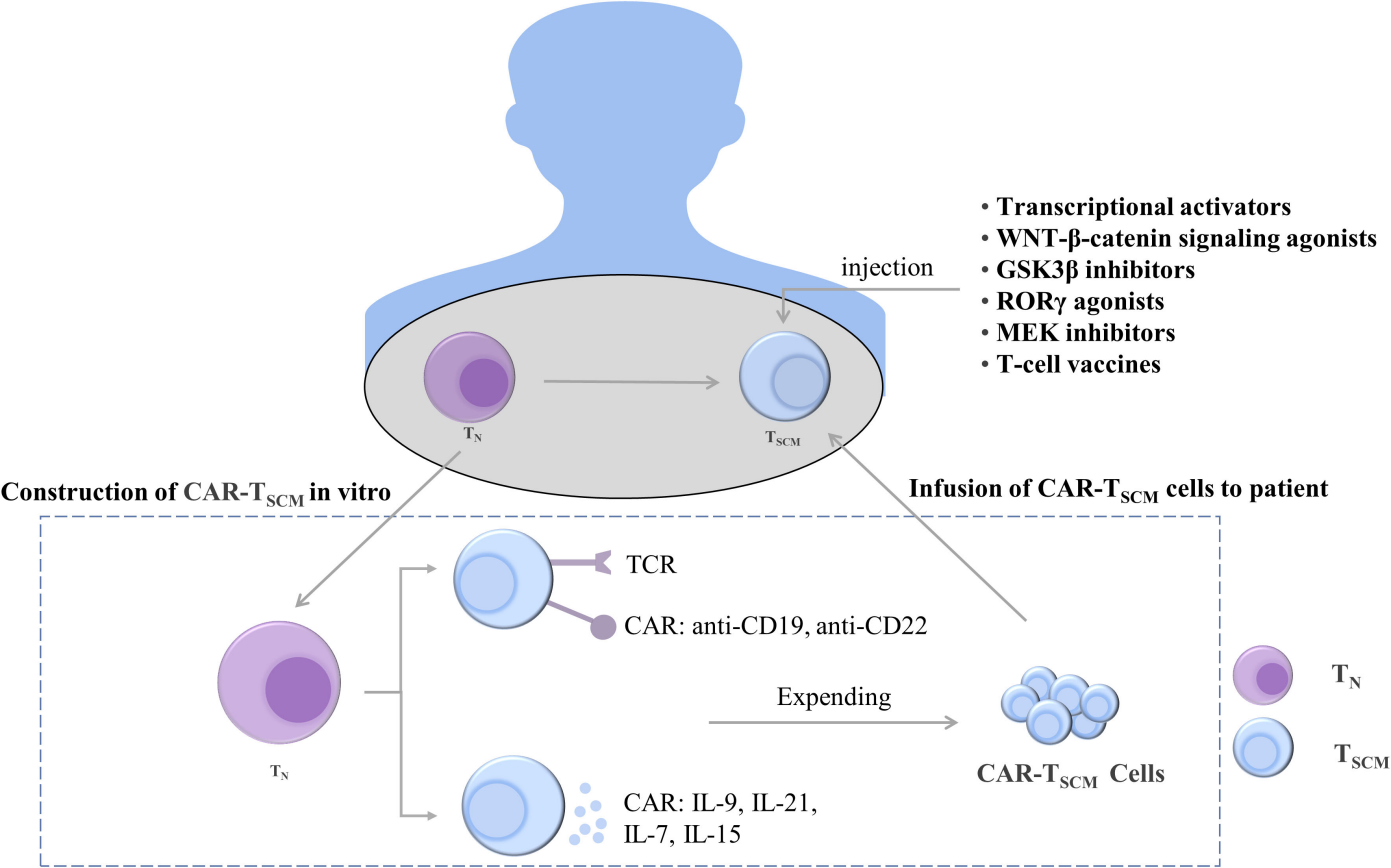
Treatments to maintain and induce T cell stemness. T cells can be constructed *in vivo* into CAR-T_SCM_ cells expressing anti-CD19 and anti-CD22 and CAR-T_SCM_ cells expressing cytokines IL-9, IL-21, IL-7, and IL-15. The stemness-enhancing CAR-T_SCM_ cells can be infused back into patients for tumor treatment. In addition, enhanced T cell stemness can also be induced by using transcriptional activators, WNT-β-catenin signaling pathway agonists, GSK3β inhibitors, RORγ agonists, MEK inhibitors, and T cell vaccines. T_N_, naïve T cells; T_SCM_, stem cell-like memory T cells; IL, interleukin; TCR, T-cell receptor; CAR, chimeric antigen receptor; GSK-3β, glycogen synthase kinase-3β; RORγ, Retinoic acid receptor-related orphan receptor γ; MEK, mitogen-activated protein kinase.

T_SCM_ have stem cell-like properties and strong differentiation potential. T_SCM_ and T cells both increased in patients with HIV after antiretroviral therapy (200). Stem cell-like T cells express CD62L, CCR7, TCF1, and LEF1 and can rapidly secrete IFN-γ, IL-2, and TNFα (201, 202). Increasing the expression of stemness-related genes can induce pluripotent stem cell-like T cells. Thus, the genetic reprogramming of differentiated T cells into less differentiated cells can generate a T cell-specific stemness phenotype (**Figure 5**).

T cells expressing TCF1 were identified as a group of cells with T-cell stemness; they possess the ability to undergo exponential expansion and self-renewal and differentiate into tumor-infiltrating lymphocytes. Intravenous injection of nanoparticle-based vaccines can generate more stem cell-like TCF1^+^ T cells, and the number of TCF1^+^ T cells in patients was positively correlated with the treatment outcome (99). Ablation of TCF1 suppressed the immunotherapy response (203). c-Myb is a transcriptional activator of TCF1, and overexpression of c-Myb in B16 tumor mice enhanced the multifunctionality of CD8^+^ T cells and production of T_SCM_ as well as promoted the antitumor effect (204). The maintenance of T-cell stemness is dependent on WNT-β-catenin signaling, and WNT3A or GSK3β inhibitors promote stemness-related gene expression in T_SCM_ (115, 205). When the expression of β-catenin is stabilized, T cells differentiate into effector T cells, inhibiting the stem cell-like characteristics of T cells and weakening the differentiation ability of T cells. The use of RORγ agonists increased the stemness of Th17 cells (206). In addition, selumetinib, an MEK1/2 inhibitor (MEKi), promoted the generation of T_SCM_ by inhibiting the MAPK pathway, retarding cell cycle progression, and enhancing metabolic fitness. Moreover, MEKi treatment in tumor-bearing mice resulted in a strong antitumor effect (165). The enhancement of the stemness of T cells is helpful for treating tumors, and its therapeutic effect has been demonstrated. However, additional mechanisms through which the stemness of T cells is maintained remain to be identified.

#### 4.2.2 Enhancement of stemness characteristics of T cells by CAR-T therapy

Adoptive transfer of T-cell therapy is an emerging cancer treatment modality that involves genetically engineering a patient’s T cells to generate T cells that target cancer cells and are then infused back into the patient to exert an antitumor effect. Among them, CAR-T cell therapy is widely used in clinical practice, and it exerts a cytotoxic effect by binding to specific antigens on the surface of cancer cells (207). IL-9-secreting (T9) CAR-T cells have an enhanced T stem cell-like phenotype and can differentiate into effector T cells (208). CAR-T cells can secrete soluble molecules that activate T-cell stemness. For example, IL-21 maintains T-cell stemness and survival by inhibiting the formation of T_EFF_ (148). The inoculation of CAR-T cells expressing IL-15 into mouse tumors promoted the upregulation and maintenance of TCF1 and increased the proportion of stem cell-like memory T cells. Furthermore, the inoculation of CAR-T cells coexpressing IL-15 and IL-21 in mice resulted in more robust T-cell expansion compared with IL-15 CAR-T treatment (209). In addition, IL-7 can stimulate the proliferation of T_SCM_. When CAR-T cells secreting IL-7 and chemokine (C–C motif) ligand 19 were intravenously injected into patients with advanced liver cancer, the tumors almost completely disappeared (210). These studies indicate that CAR-T cells, which secrete cytokines to enhance the stemness of T cells, exhibit significant antitumor activity in solid tumors.

More T_SCM_ can be produced by enhancing the effect of CAR-T cells, which can increase the antitumor effect. Regnase-1 deficiency can reduce TCF1 CAR-T cell fatigue, indicating that CAR-T cells support the expansion and the formation of T_SCM_ (211). Furthermore, the blockade of the BET bromodomain with JQ1 downregulated BATF expression to preserve the T_SCM_-enriched transcriptome signature, and CAR-T cells exhibited enhanced proliferative capacity (212). The addition of Cas-CLOVER, a high-fidelity nuclease, to CAR-T cells led to the production of stable T_SCM_ exhibiting robust antitumor activity in both *in vitro* and *in vivo* models (213). Decitabine can be used to enhance the stemness of CAR-T cells. Compared with untreated CAR-T cells, CAR-T cells treated with low-dose decitabine exhibited higher expression of the T-cell stemness–related genes LEF1, TCF7, and Bcl6 and had better T-cell expansion ability (214). Construction of the allogeneic MUC1-C-specific CAR-T cells P-MUC1C-ALLO1, which were used to treat breast and ovarian cancer through xenotransplantation, exerted an effective curative effect *in vivo* (209). Wei et al. reported that the simultaneous activation of the costimulatory signals 4-1BB and DAP10 on NKG2D CAR-T cells resulted in the poor differentiation of T cells *in vitro* and *in vivo*, upregulation of Bcl-2 expression, and an increased proportion of T_SCM_ subsets (215). CAR-T cell adoptive transfer therapy results in significant antitumor activity, but this effect is observed only in a small group of patients. By enhancing the stemness of T cells, CAR-T cells may produce stronger antitumor properties. These studies provide a strong scientific basis for the rapid development of T_SCM_ in human clinical trials of adoptive immunotherapy.

#### 4.2.3 Enhancement of the stemness characteristics of T cells by cancer vaccines

Tumor vaccines are currently under development. Cancer therapeutic vaccines use peptides to mimic cancer epitopes presented by major histocompatibility complex molecules in combination with potent adjuvants to induce T_SCM_. Cancer vaccines control and eliminate tumors by generating robust and durable antitumor responses while overcoming immune tolerance and immunosuppression. Currently, studies on T-cell vaccines are conducted enrolling only a small number of patients. Serial vaccination with the Melan-AMART-126-35 peptide, CpG-B, and complete Freund adjuvant stably expanded tumor-specific CD8^+^ T_SCM_ cells after 6 months and supported long-term antitumor immunity (216). Therefore, tumor vaccines are used to enhance the anti-tumor effects of T cells by inducing the expression of T_SCM_. However, knowledge of cancer vaccines is inadequate. To substantially induce T_SCM_, the selection of antigen targets, the efficacy of adjuvants, and the optimization of drug delivery systems are crucial; these need to be explored in future studies. Cancer vaccines are a potential direction of immunotherapy.

## 5 Conclusion and perspectives

T cells play a crucial role in killing tumors in the TME. However, because of the immunodeficiency of T cells and the negative regulation of T cells by the TME, tumor progression cannot be optimally controlled currently. In this review, we discussed the characteristics of T_EX_ and stem cell-like T cells to improve our understanding regarding them. T-cell exhaustion is a state of disability, which includes T-cell expression inhibitory immune checkpoints and cytokine secretion disorder. According to the characteristics of T-cell exhaustion, immune checkpoint inhibitors and soluble mediators can be used to reverse the exhaustion of T cells, thus enhancing the antitumor effect of T cells. T_SCM_ have unique stemness characteristics, long-term self-renewal ability, and pluripotency, and maintaining the stemness of T cells has become the focus of current research. The induction and expansion of T_SCM_ *in vitro* and infusing them into patients can produce a stable antitumor effect *in vivo*, which is the main goal of CAR-T therapy. We summarized the characteristics and mechanisms of action of T_EX_ and stem cell-like T cells as well as discussed current therapies aimed at reversing T-cell exhaustion and supporting T-cell stemness to maintain durable T-cell responses. These methods have demonstrated effectiveness in the treatment of tumors. However, the specific mechanisms of action and related therapeutic regimens in T_SCM_ and T-cell exhaustion need to be further explored to fully understand immune and molecular changes during the whole treatment process and to predict drug efficacy and possible recurrence. Efficient and persistent T-cell action can ensure the long-term prevention and treatment of cancer and infections.

## Author contributions

XC, SL and PY wrote and edited the draft. W-LH and reviewed and edited the manuscript. W-HY, XY and J-HC wrote the manuscript and supervised the entire work. All authors agree to be responsible for the publication. All authors contributed to the article and approved the submitted version.
